# Supplementary material for: Challenges Pertaining to the Optimization of Therapy and the Management of Asthma—Results from the 2023 EU-LAMA Survey
Source: Biomedicines. 2025 Aug 1;13(8):1877. doi: 10.3390/biomedicines13081877 (PMC12383591; doi:10.3390/biomedicines13081877)
Supplement: Supplementary file 1 [file biomedicines-13-01877-s001.zip › Online_repository.pdf]

Statistical details regarding the physicians who participated in the EU-LAMA Survey

Physicians from all participating countries, including general practitioners, allergologists, pulmonologists, internal medicine specialists, and medical physicians in specialization, were invited to participate in this research. Information concerning the workplaces of these physicians was collected: physician's professional practice [hospital (ward + specialist hospital policlinic/ambulatory), specialist hospital policlinic and general policlinic (private/public health center)]. Information regarding the size of the medical centers in which the physicians were employed was also obtained: city with up to 50,000 residents or countryside; city with between 50,000 and 150,000 residents; city with between 150,000 and 500,000 residents; and city with more than 500,000 residents. Among the 767 surveys that were opened, 630 were fully completed, and only these surveys were included in the data analysis.

The survey was conducted anonymously, and access to the survey link was restricted through a random unique access code, which was provided to physicians by the Chiesi Poland Medical Department.

The study was conducted from April to December 2023; in particular, the first survey was collected on April 6th, 2023, and the final survey was completed on December 21st, 2023. Representatives of the Chiesi Poland Medical Department oversaw the project's execution and provided physicians with support in terms of survey access; the CRO was responsible for the methodology, study tool design, and data analysis.

The analysis focused on assessing therapeutic undertreatment among patients who should be managed at GINA Step 5

This issue is often associated with key clinical decisions, such as whether to add a LAMA to current treatments, to increase the dose of ICS to the recommended maximum, to

initiate the use of oral corticosteroids (OCS), or to refer patients for biological treatment. Despite the availability of these options, physicians frequently continue to treat patients at GINA Step 4, even when their condition clearly necessitates escalation to Step 5.

Therapeutic underestimation in the context of asthma analysis was based on four specific responses to survey questions 6 (which pertains to patients with asthma at GINA Step 4) and 7 (which pertains to patients with asthma at GINA Step 5). This study aimed to compare the real-world frequency of the use of certain treatment methods among patients with severe asthma to the treatment guidelines outlined in the GINA 2023, thus revealing potential therapeutic underestimation from the perspective of patients.

Real-world treatment data were obtained directly from the survey, specifically from the responses to question 7, which reflect the actual treatment practices reported by the participants. On the other hand, the data obtained from the GINA 2023 guidelines were extracted from the questionnaire on the basis of a methodology that suggested that if researchers employ any of the treatment strategies listed in the table for patients with asthma at GINA Step 4, these patients should theoretically be classified under GINA Step 5 rather than remaining in their previous category.

To create the guideline-based treatment dataset, selected responses to question 6 were analyzed. The use of these methods among patients at GINA Step 4 was then combined with participants' responses to question 7. To explain this process in further detail, let us consider responses to the topic "To add LAMA to current treatment" more closely:

- If the researcher answered both questions 6 and 7 affirmatively, the patient was included in both datasets as using the treatment method in question.
- If the response to question 6 was affirmative but the response to question 7 was negative, the patient was not included in the real-world treatment dataset but was included in the guideline-based treatment dataset.

- If the response to question 6 was negative but the response to question 7 was affirmative, the patient was included in both datasets as using the treatment method in question.

Table presents the method used to create both datasets.

Table S1. Inclusion criteria used for the real-world and guideline-based treatment datasets.

Response to Question 6	***Response to Question 7***	**Included in the Real-World Treatment (Question 7)**	**Included in the Guideline-Based Treatment (Question 7 + Affirmative Responses from Question 6)**
1	1	Yes	Yes
1	0	No	Yes
0	0	No	No
0	1	Yes	Yes

The counts pertaining each category, alongside the corresponding percentages, were calculated on the basis of the prepared datasets. To compare real-world treatment with guideline-based treatment, McNemar's test was performed. This method was chosen because the data were paired, such that each subject contributed responses under both conditions. Unlike the chi-square test or Fisher's exact test, McNemar's test is specifically designed to account for dependency between paired observations, thereby ensuring an accurate analysis of the differences.

McNemar's test is commonly used to analyze categorical outcomes (e.g., yes/no or success/failure) for the same individuals across two time points or treatment approaches. In this analysis, the test was employed to determine whether significant differences existed between the treatments provided in the real-world setting and those recommended by the guidelines. By focusing on discordant pairs—i.e., cases in which a patient’s treatment differed

between the two conditions—McNemar's test can be used to determine whether these discrepancies are statistically significant. This approach helps researchers evaluate whether the differences observed between real-world and guideline-based practices are meaningful or could rather be attributed solely to chance.
