# Supplementary material for: Challenges Pertaining to the Optimization of Therapy and the Management of Asthma—Results from the 2023 EU-LAMA Survey
Source: Biomedicines. 2025 Aug 1;13(8):1877. doi: 10.3390/biomedicines13081877 (PMC12383591; doi:10.3390/biomedicines13081877)
Supplement: Supplementary file 1 [file biomedicines-13-01877-s001.zip › Supplementary Figures.pdf]

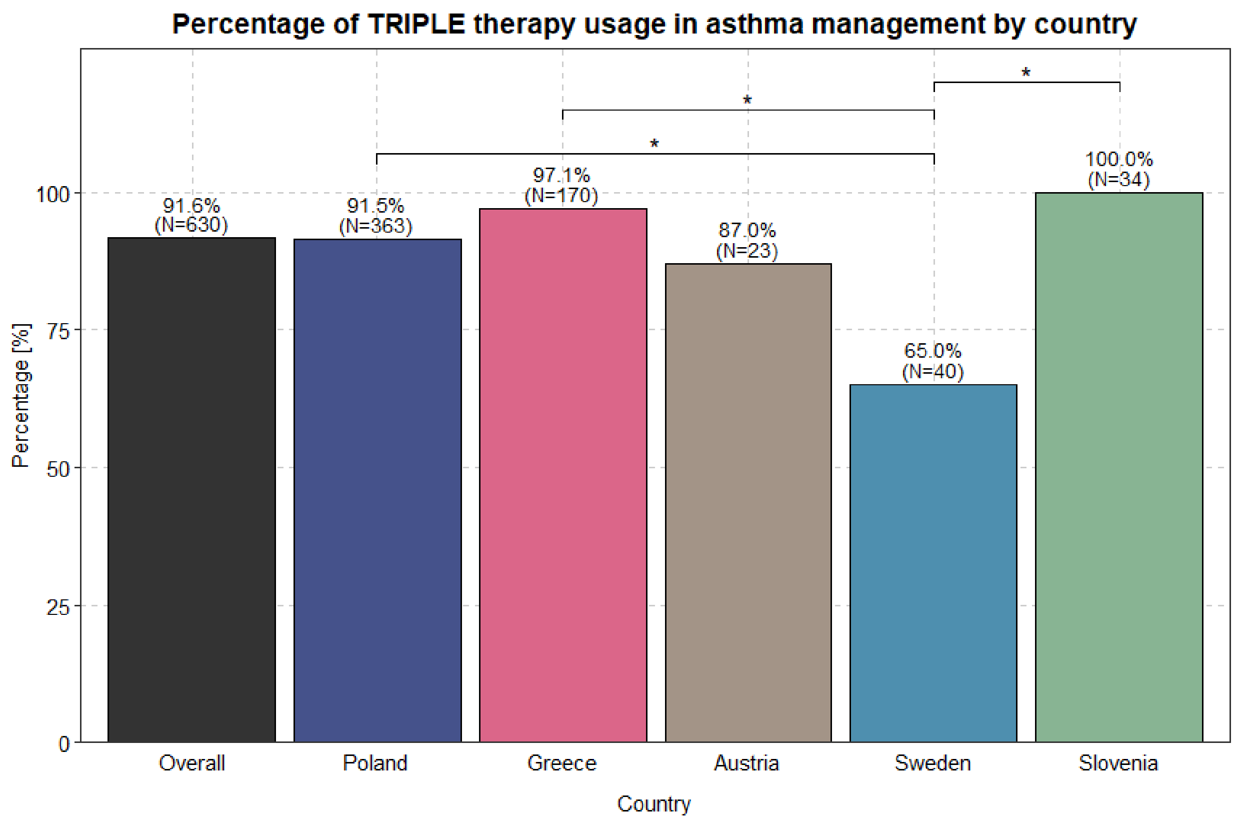

Figure S1. Percentage of triple therapy (ICS + LABA + LAMA) usage in asthma management by country.

The asterisk ("*") indicates statistically significant differences between groups ($p < 0.05$).

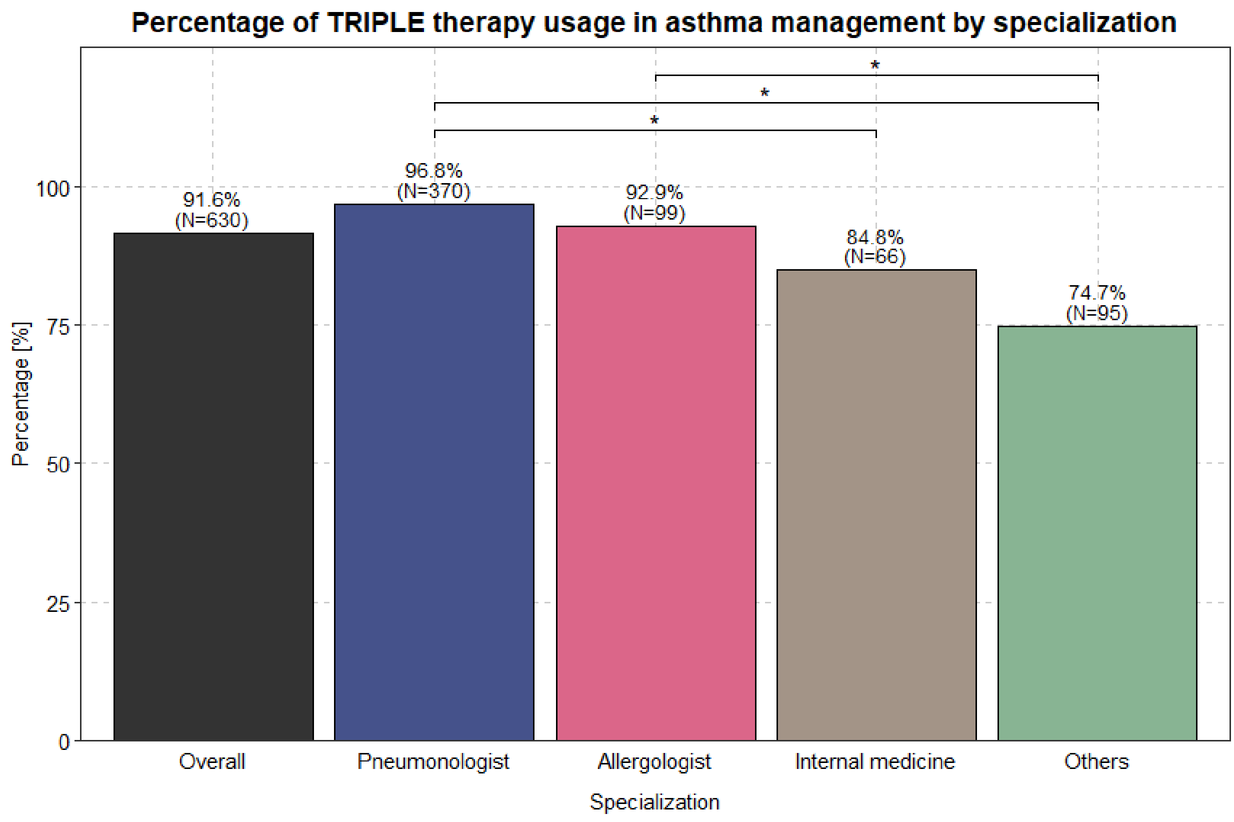

Figure S2. Percentage of TRIPLE therapy (ICS + LABA + LAMA) usage in asthma management by specialization.

The asterisk ("*") indicates statistically significant differences between groups ($p < 0.05$).

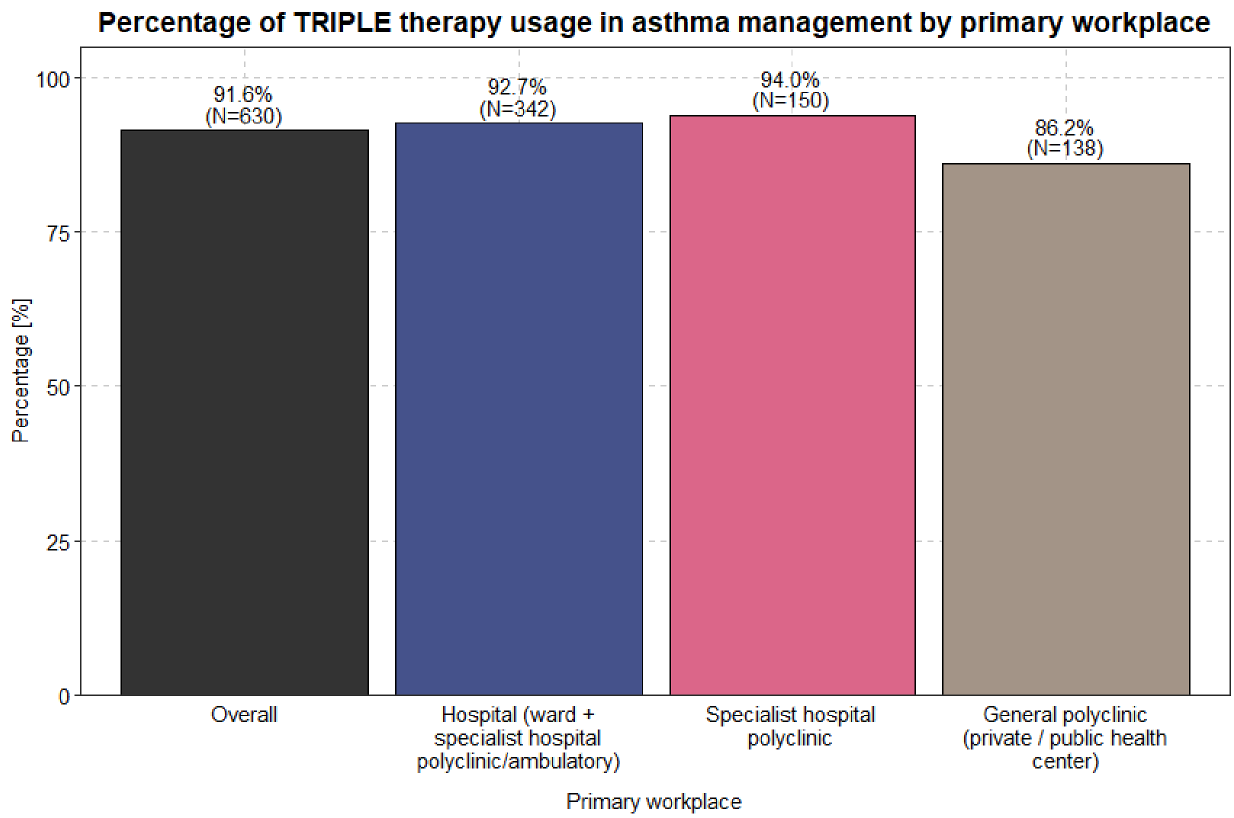

Figure S2. Percentage of TRIPLE therapy (ICS + LABA + LAMA) usage in asthma management by primary workplace.

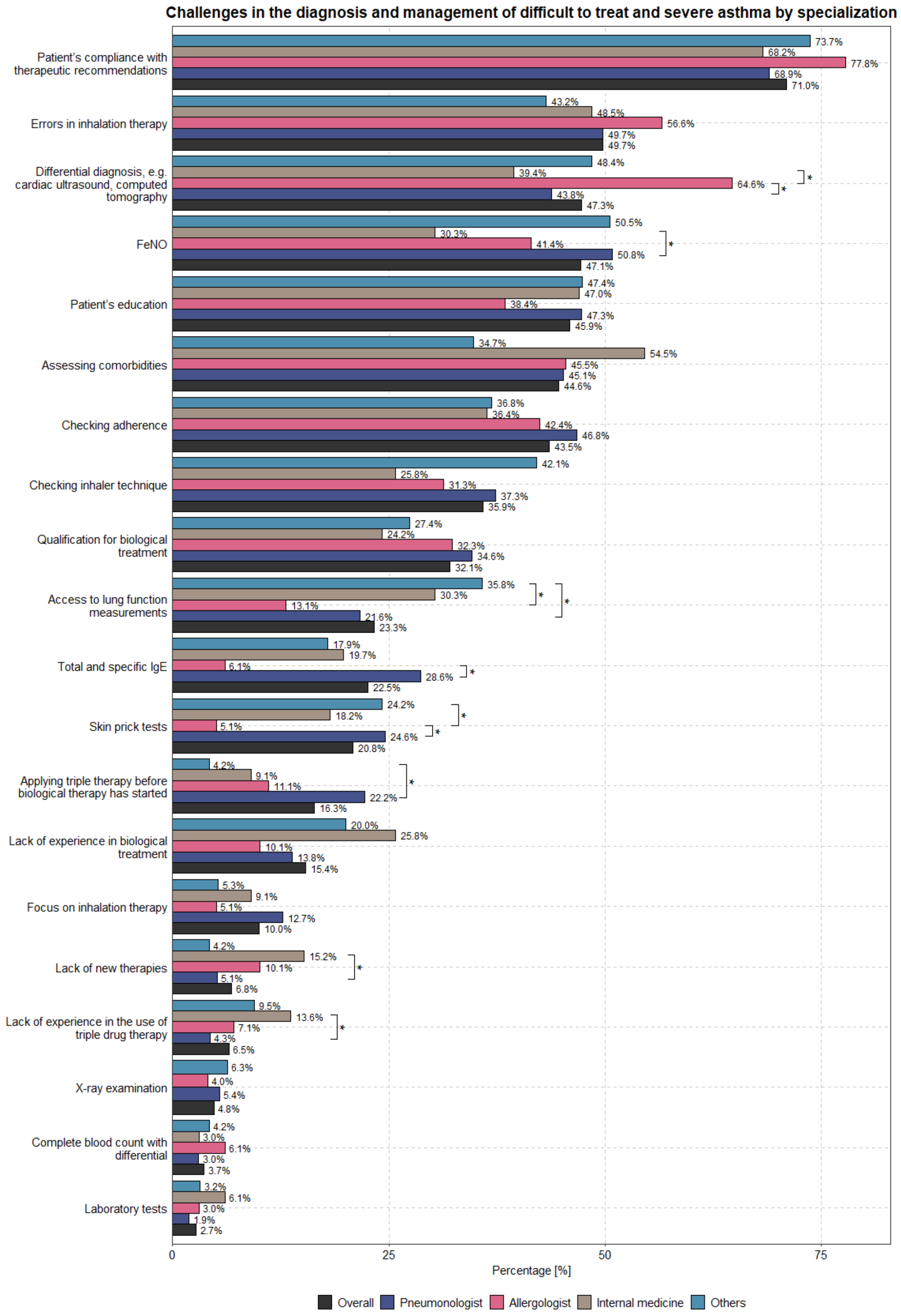

Figure S4. Challenges in the diagnosis and management of difficult to treat and severe asthma by specialization.

The asterisk ("*") indicates statistically significant differences between groups ($p < 0.05$).

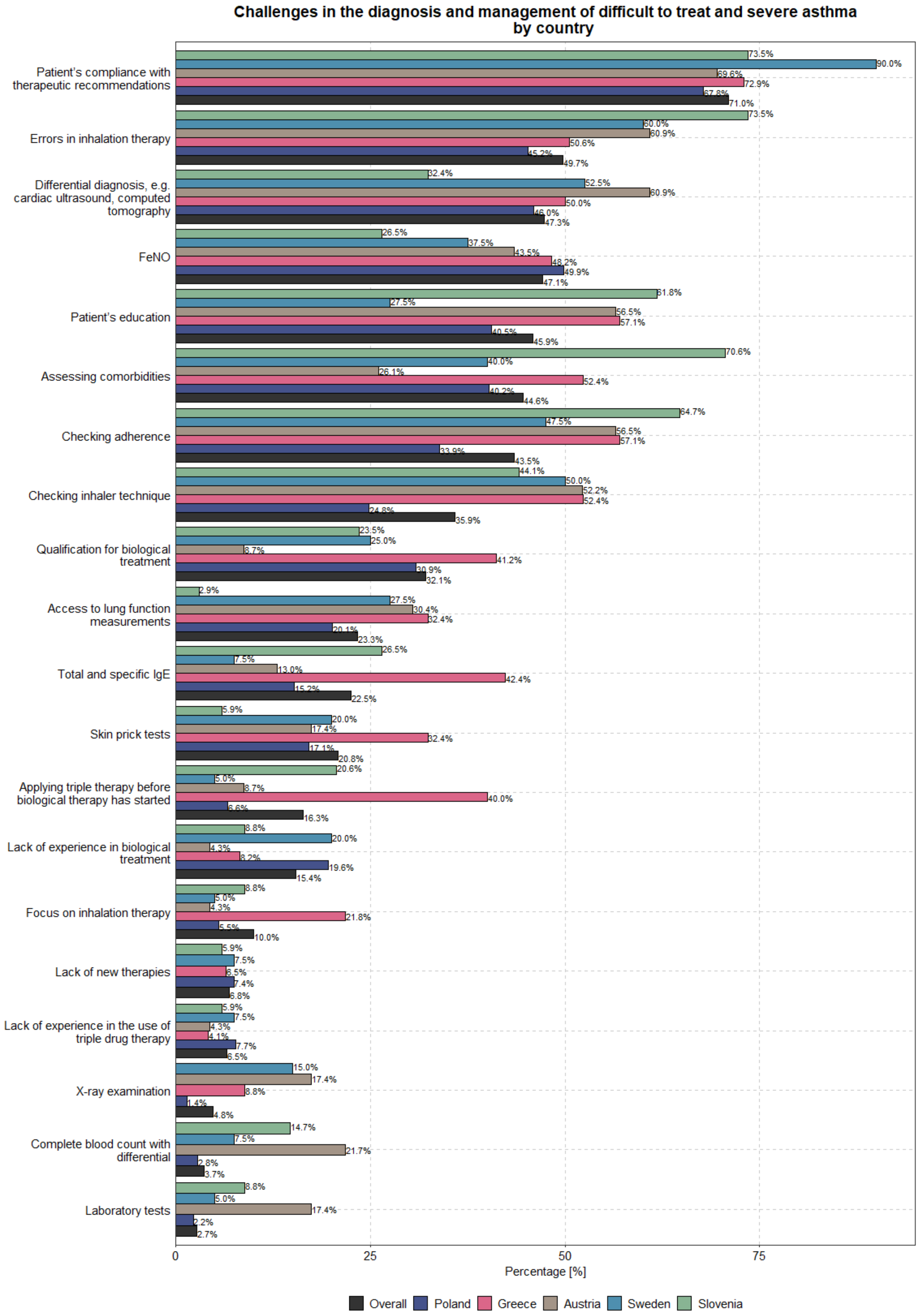

Figure S5. Challenges in the diagnosis and management of difficult to treat and severe asthma by country.

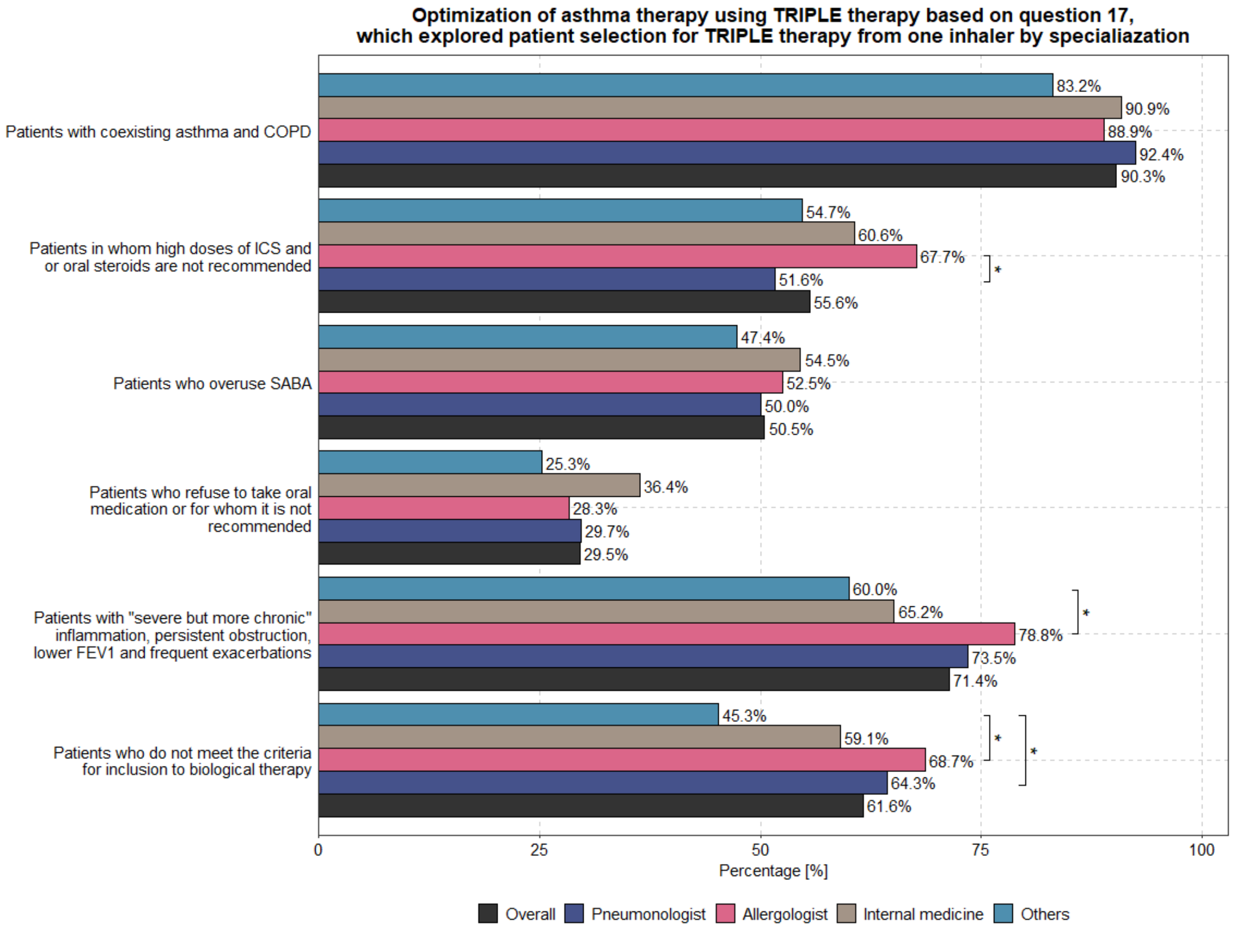

Figure S6. Optimization of asthma therapy using triple therapy by specialization.

The asterisk ("*") indicates statistically significant differences between groups ($p < 0.05$).

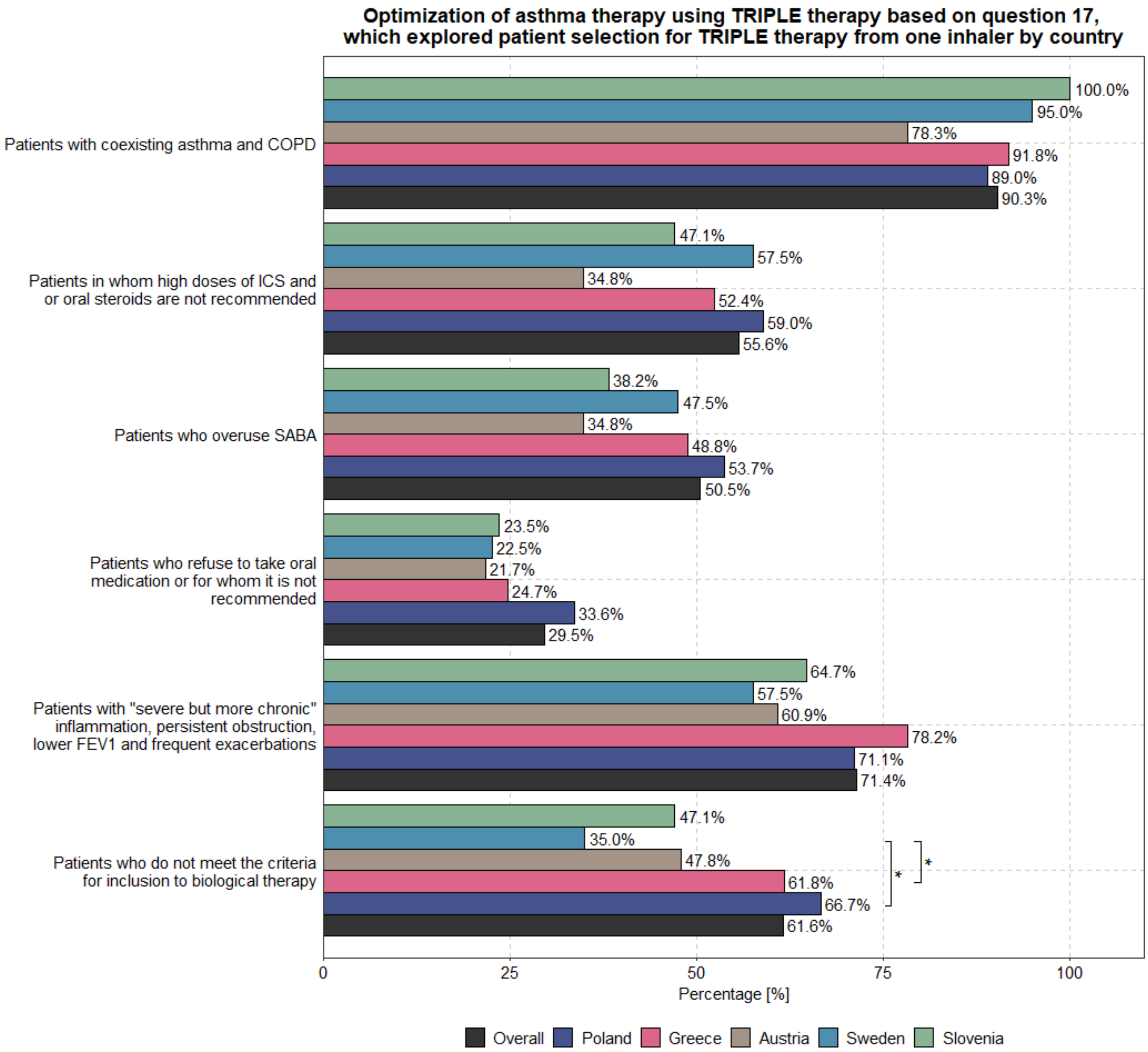

Figure S7. Optimization of asthma therapy using triple therapy by country.

The asterisk ("*") indicates statistically significant differences between groups ($p < 0.05$).

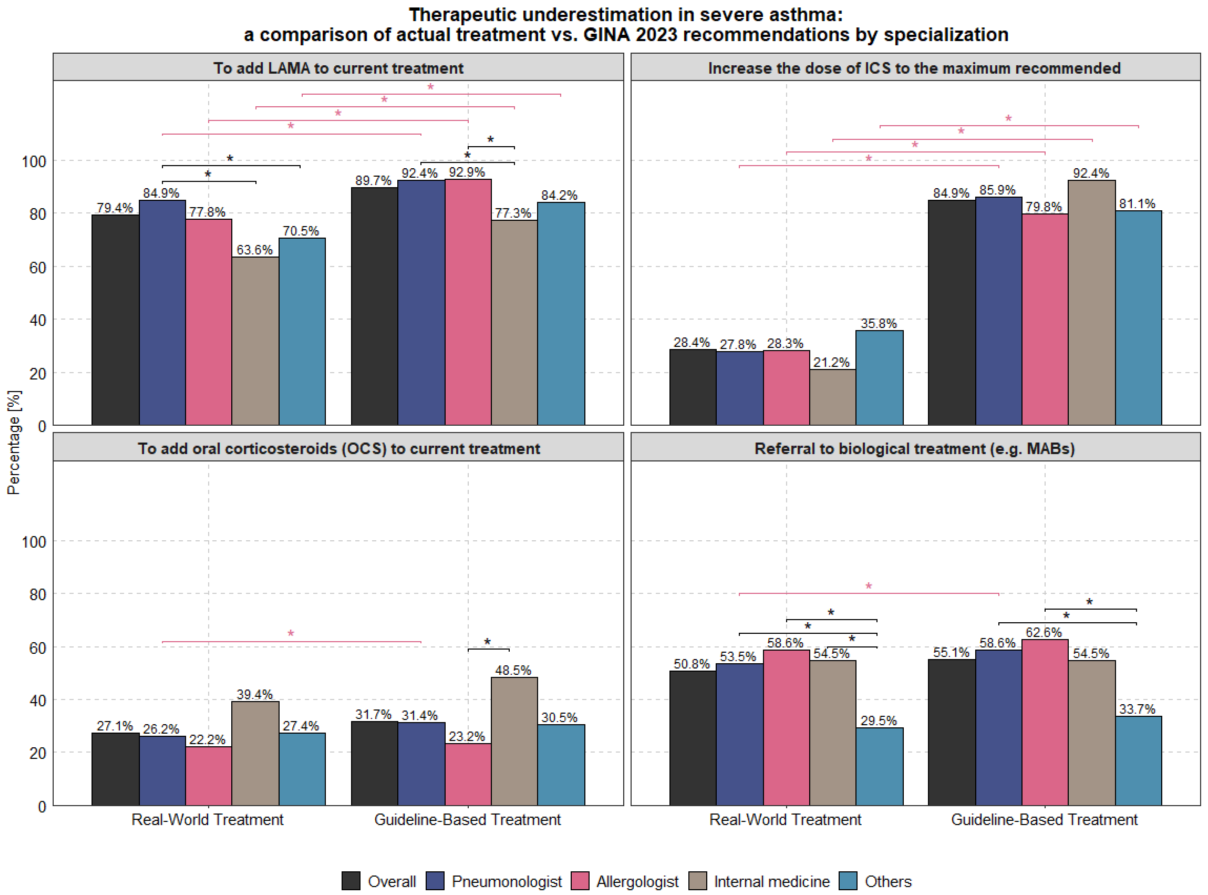

Figure S8. Therapeutic underestimation in severe asthma: a comparison of actual treatment *vs* GINA 2023 Recommendations by specialization.

The asterisk ("*") indicates statistically significant differences between groups ($p < 0.05$).

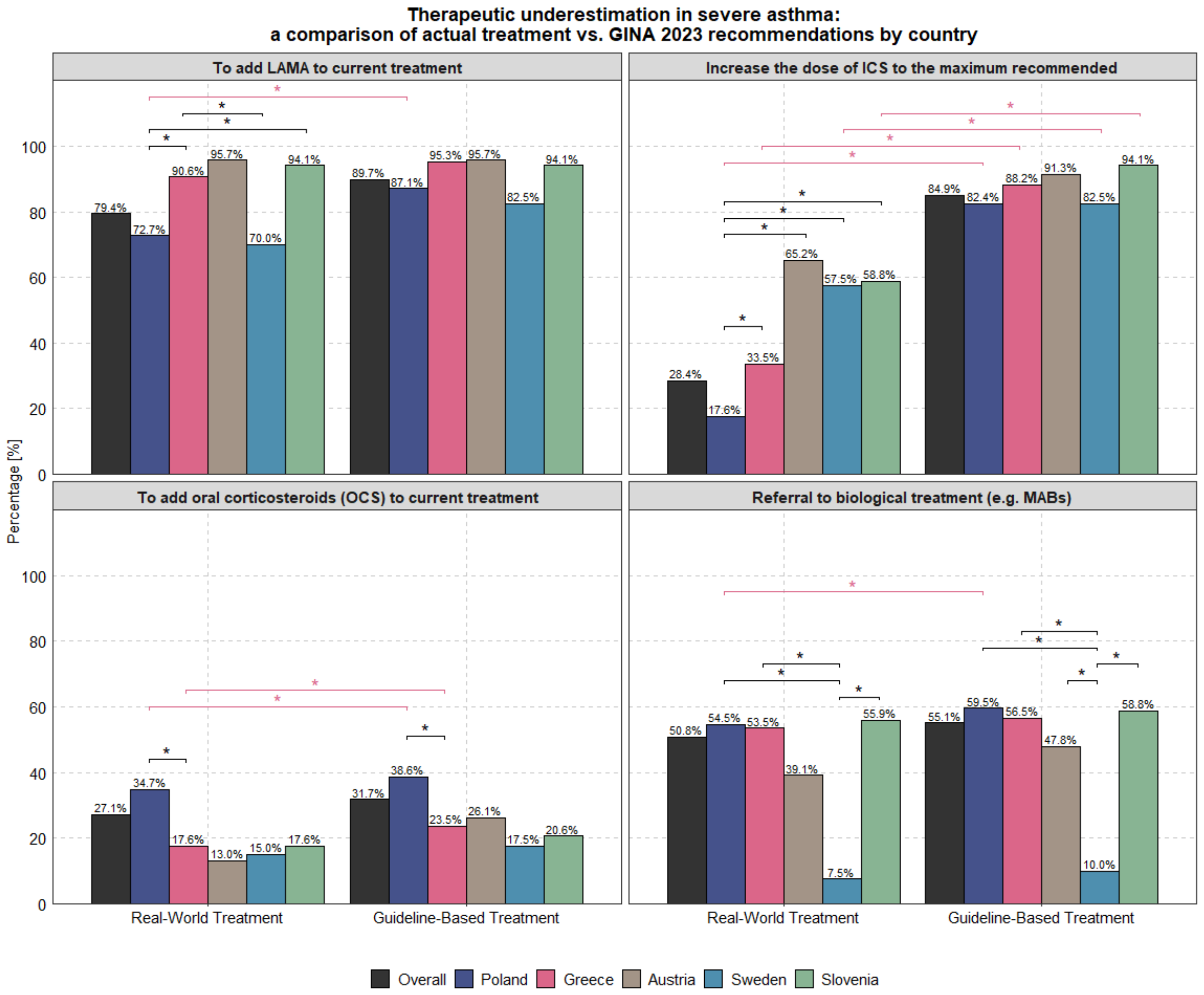

Figure S9. Therapeutic underestimation in severe asthma: a comparison of actual treatment *vs* GINA 2023 Recommendations by country.

The asterisk ("*") indicates statistically significant differences between groups ($p < 0.05$).
